# Modulating Heat Input to Optimize Corrosion Resistance of Nickel–Aluminum Bronze Manufactured by Cold Metal Transfer Additive Manufacturing

**DOI:** 10.3390/ma18102205

**Published:** 2025-05-10

**Authors:** Renjie Huo, Zheying Wang, Mingsheng Wang, Rui Wang, Song Zhang, Chunhua Zhang, Chenliang Wu, Haitao Chen, Jiang Chen

**Affiliations:** 1 School of Materials Science and Engineering, Shenyang University of Technology, Shenyang 110870, China; rjhuo88@163.com (R.H.); zhangch5858@126.com (C.Z.); mfclwu@163.com (C.W.); 2 School of Materials, Wuzhou University, Wuzhou 543003, China; 3 Institute of Metal Research, Chinese Academy of Sciences, Shenyang 110016, China; mswang@imr.ac.cn; 4 Benxi Tools Co., Ltd., Benxi 117004, China; wangrui4836@163.com; 5 Shenyang Dalu Laser Technology Co., Ltd., Shenyang 110136, China; htchenlaser@163.com (H.C.); jchenlaser@163.com (J.C.)

**Keywords:** additive manufacturing, cold metal transfer, microstructure evolution, corrosion

## Abstract

The influence of heat input (HI) on the microstructure, microhardness, electrochemical corrosion performance of cold metal transfer additively manufactured (CMTAM) nickel–aluminum bronze alloys was investigated. The nickel–aluminum bronze exhibited an α-Cu austenite matrix with minor γ_2_-Cu_9_Al_4_ and κ phases. As HI increased, the microstructure coarsened progressively. Electron backscatter diffraction (EBSD) analysis revealed that with increasing HI, the grain size gradually increased and the Schmid factor increased. Consequently, the microhardness declined from 198.3 HV to 171.7 HV. The decrease in microhardness with increasing heat input is primarily attributed to the grain coarsening and the coarsening and uneven distribution of the κ phase. As the heat input (HI) increased from 243.8 J/mm to 644.7 J/mm, the corrosion current density rose significantly from 2.56 ± 0.04 μA/cm^2^ to 7.52 ± 0.07 μA/cm^2^. This result indicates a marked deterioration in the material’s corrosion resistance. This phenomenon can be attributed to the grain coarsening and the distribution of Al solute within the microstructure. The CMTAM nickel–aluminum bronze alloys hold significant potential for enhancing the reliability and long-term protection of marine engineering equipment.

## 1. Introduction

Nickel–aluminum bronze (NAB) alloy is extensively suitable for shipbuilding industry and marine engineering components, including propellers, ball valves, butterfly valves, piping systems, and pump bodies, owing to its superior resistance to seawater corrosion, wear corrosion, erosion corrosion, cavitation corrosion, and biofouling [1,2,3]. As implied by its name, NAB primarily incorporates aluminum as the principal alloying element. At elevated temperatures, aluminum forms a solid solution within the face-centered cubic copper matrix. During cooling, the κ phase precipitates from the α-Cu matrix, resulting in precipitation strengthening. The presence of aluminum facilitates the formation of a dense Al_2_O_3_ oxide film on the surface, which significantly contributes to high corrosion resistance of the alloys. However, elevated aluminum concentrations have been observed to promote precipitation of thermodynamically stable β-phase intermetallic compounds at high temperatures, which undergoes a eutectoid transformation at 565 °C, producing α + γ_2_ phases [4,5]. γ_2_ phase is a brittle phase that adversely affects mechanical properties. Meanwhile, the formation of the γ_2_ phase will lead to a significant decrease in the corrosion resistance. To mitigate these issues, nickel is added to improve corrosion resistance and prevent slow-cooling brittleness in thick castings; iron is included to refine grain structure and enhance mechanical properties; and manganese is incorporated to improve melt fluidity and microstructure [6]. Among NAB alloys, high nickel–aluminum bronze C63280 (AWS A5.7 compliant) demonstrates extensive applications in offshore engineering and naval construction among naval bronze alloys, owing to its superior resistance to marine corrosion and mechanical abrasion. The corresponding grade is ERCuNiAl. The solidification process of NAB involves complex solid-state phase transformations. The cast alloy primarily consists of Cu-rich α phase, residual β phase, and κ phases [7]. Rapid solidification processes such as laser cladding and additive manufacturing result in a microstructure comprising α-Cu phase, β′ martensite, γ_2_, and κ phases [8]. In some non-equilibrium weld microstructures, the β′ martensite phase may be absent [9]. Previous studies have demonstrated that heat treatment [10], in situ alloying [11], friction stir processing [2], and other methods can enhance mechanical and corrosion performance of NAB. Therefore, investigating the phase composition and evolution of NAB and improving its comprehensive performance are crucial for expanding its applications.

Given the intricate manufacturing processes and significant time and economic costs associated with large components, such as marine propellers, and critical equipment, such as heat exchangers and condensers, remanufacturing has become a preferred approach. This method not only expedites the provision of functional components but also enhances the original components by addressing defects and performance limitations, thereby improving structural integrity and extending service life. Remanufacturing techniques encompass mechanical processing methods (e.g., turning, milling, grinding), surface engineering technologies (e.g., thermal spraying, electroplating, laser cladding), and additive manufacturing. Additive manufacturing offers advantages such as high material utilization, precise repair accuracy, and customized solutions. Technologies like laser additive manufacturing [12], electron beam additive manufacturing [13], and arc welding additive manufacturing have seen widespread application. Over the past few years, CMTAM has emerged as a focus of research due to its low dilution rate, high efficiency in production, and cost-effectiveness.

The CMTAM process effectively mitigates metallurgical defects caused by high heat accumulation in traditional arc additive manufacturing through precise control of droplet transfer and heat input. This technology offers an innovative solution for fabricating metal components with fine microstructures and superior mechanical properties. The low dilution rate inherent in CMT significantly reduces the impact of subsequent deposition layers on previous ones, which is a critical factor contributing to the widespread attention CMTAM has garnered. Research indicates that the unique “cold–hot alternating” thermal cycle mode of CMTAM exhibits pronounced temperature mitigation effects in liquid-phase processing and refine grain size. These characteristics provide CMTAM [14,15,16] with distinct advantages in high-end manufacturing fields such as aerospace precision components, marine engineering parts, metallurgical machinery remanufacturing, and biomedical implants, making it an important research direction in intelligent arc additive manufacturing. Scholars have extensively studied the CMT additive manufacturing of nickel–aluminum bronze. Dharmendra et al. [17] Performed microstructural analysis of the fabricated material compared phase formation differences relative to cast nickel–aluminum bronze (NAB) based on solidification characteristics. In WAAM-NAB, the high cooling rate suppressed κ_I_ phase formation, resulting in a finer microstructure and a significant reduction in intermetallic particle volume fraction. EDS and TEM diffraction pattern analysis revealed that the phases formed between dendrites were spherical Fe_3_Al and lamellar NiAl. Fe-enriched κ_IV_ precipitates were homogeneously dispersed throughout the α-phase matrix. Crystallographic orientation mapping via EBSD revealed limited orientation preferences along both depositional axes, with <5% maximum pole density observed in the (100) directionally rotated specimens. Additively manufactured nickel–aluminum bronze (NAB) components exhibited a statistically significant enhancement in mechanical properties, with yield strength and elongation values averaging 88 MPa and 10%, respectively, surpassing their cast counterparts by 22.5% and 33.6% in corresponding parameters, although the increase in ultimate tensile strength was relatively limited. Aliyu and co-workers [18] demonstrated that post-deposition annealing substantially optimized the metallurgical characteristics of nickel–aluminum bronze through refinement of microstructural uniformity, controlled grain growth, and optimized κ-phase dispersion. This thermal processing concurrently alleviated internal stress concentrations and induced preferential development of Σ3–60°//[111] low-energy grain boundaries. The resultant microstructural evolution augmented corrosion resistance in chloride-rich environments (3.5% NaCl), mitigated localized corrosion susceptibility, and fostered the development of protective oxide layers. Comparative analysis revealed thermally processed NAB specimens demonstrated markedly improved corrosion resistance and extended service longevity relative to their non-treated counterparts. Shen et al. [19] conducted morphological characterizations on additively manufactured components subjected to various post-heat treatments. Experimental results indicated that both longitudinal and transverse strengths of the deposited structures exceeded normal strength. By applying a designed quenching and tempering method, the ductility of the alloy was significantly enhanced, and at a tempering temperature of 650 °C, the alloy demonstrated superior mechanical properties. Additionally, quenching–tempering treatment effectively alleviated crystallographic anisotropy in the additively manufactured (AM) alloy. To sum up, it is evident that microstructural features significantly influence the mechanical and corrosion properties. Heat input is a key factor directly affecting grain size, phase transformation, and microstructure morphology during the additive manufacturing process. This study employed the CMTAM process method to fabricate C63280 high-nickel–aluminum bronze alloy, systematically investigating the influences of different heat inputs on the microstructure, microhardness, and corrosion resistance of the additively manufactured structures. Thus, it provides important reference value for practical applications in the field of marine engineering.

## 2. Experiments and Methods

### 2.1. Materials and Specimen Fabrication

NAB was fabricated on the surface of a 6 mm thick copper substrate using an additive manufacturing process. The welding power supply employed was the Advanced 4000R CMT system (Fronius company, Graz, Austria), and the material used was a commercially available high-nickel–aluminum bronze ERCuNiAl wire with a diameter of 1.2 mm. The detailed chemical compositions of both the wire and the substrate are provided in Table 1. Prior to the experiment, the substrate underwent ultrasonic cleaning in high-purity acetone (99.9%) for three cycles, followed by a 15 min immersion process to effectively eliminate residues and subsequently dried for use. During the welding process, an integrated optimization control system ensured precise coordination between welding parameters and process requirements. The deposition path followed a reciprocating motion along the substrate direction, which effectively ensured uniform forming quality at both ends of the sample. The lap joint ratio of the additive manufacturing alloy is 15%. Interlayer temperatures between adjacent deposited layers were strictly controlled using a handheld infrared thermometer to ensure that temperatures did not exceed 50 °C. The inert gas argon is used for gas protection, and the protection gas flow rate is 15 L/min. Specific process parameters such as current, voltage, wire feed speed (WFS), and travel speed (TS) are summarized in Table 2. Under the specified optimized conditions, potential defects such as porosity caused by low heat input and issues like deformation and spatter due to excessive heat input were minimized, thereby achieving superior formability. Heat input (HI), defined as the energy input per unit distance, was calculated within the time interval (t_1_–t_2_) using Equation [20]:

(1) $$HI=Ƞ\frac{\int_{t_{1}}^{t_{2}}IUdt}{t_{2}-t_{1}}V^{-1}$$

where U_i_ represents the instantaneous voltage; I_i_ the instantaneous current; V the welding travel speed; and η the heat transfer efficiency, typically assumed to be 0.80. In this study, an electrical signal monitoring system measured the single cold metal transition arc cycle time to be 14 ms, and the heat input is calculated based on the process parameters of a single cycle collected by the equipment and the overall process parameters of a single cladding layer, as presented in Table 2.

### 2.2. Material Characterization

Phase composition analysis of the deposited layers of the nickel–aluminum bronze were conducted using X-ray diffraction (XRD) analysis was performed using Cu Kα radiation with an operating voltage of 40 kV and a current of 30 mA. The specimens were scanned across a 2θ range of 20–100° at a scanning rate of 4°/min. For microstructural characterization, the samples underwent sequential mechanical polishing using silicon carbide abrasive papers from 320 to 2000 grit, followed by diamond paste polishing with 2.5 μm particles. Subsequently, the samples were chemically etched with a FeCl_3_-HCl aqueous solution (prepared by dissolving 5 g of FeCl_3_ in 50 mL of HCl and 100 mL of deionized water), for a duration of 8 to 10 s. The concentration of HCl is between 36% and 38%. Microstructural features, including surface morphology characteristics, were investigated using a field-emission scanning electron microscope (FE-SEM, model S-3400). The FE-SEM was equipped with energy-dispersive spectroscopy (EDS) and electron backscatter diffraction (EBSD) capabilities, enabling comprehensive microstructural characterization. This setup allowed for detailed analysis of elemental distribution and crystallographic orientation.

### 2.3. Microhardness Tests

Specimens were prepared in cubic geometries (10 × 10 × 10 mm^3^) through wire electrical discharge machining (WEDM, (Hengtian Manufacturing Co., Ltd., Taizhou, China) for subsequent characterization. Surface microhardness evaluation of the deposited layers was performed using an HVS-1000 Vickers (Dedu Precision Instrument Co., Ltd., Changzhou, China) microhardness tester under a 5 N load with 10 s dwell time. More hardness measurements were conducted for each sample, extreme results were excluded, and statistical analysis was performed on the obtained results. Prior to microhardness testing, all surfaces were sequentially ground to 2000-grit finish and polish [21,22].

### 2.4. Electrochemical Experiment

Electrochemical characterization was systematically conducted utilizing a conventional three-electrode setup in conjunction with Gamry Reference 600+ instrumentation (Gamry Electrochemistry Company, Philadelphia, PA, USA). Prior to testing, all samples were cold-mounted in epoxy resin to maintain 1 cm^2^ exposed surface area, comprising a platinum foil counter electrode, saturated calomel reference electrode (SCE), and specimen working electrode. Measurements were performed in aerated 3.5 wt% NaCl electrolyte (prepared with deionized water) under ambient conditions (25 ± 5 °C). After 3600 s of open-circuit potential (OCP) stabilization, electrochemical impedance spectroscopy (EIS) measurements were obtained within a frequency range of 10⁻^2^ to 10^5^ Hz, using a 3 mV AC perturbation signal. Potentiodynamic polarization scans were subsequently executed at 0.5 mV/s scan rate from −0.6 V to +1.3 V vs. SCE [23], preceded by 1800 s OCP monitoring to ensure interfacial equilibrium. Experimental parameters were optimized through preliminary trials to validate measurement protocols. EIS data modeling was performed using version 6.63 of Gamry Echem Analyst coupled with version 3.30 of ZsimpWin software for equivalent circuit analysis. Triplicate measurements under identical conditions ensured statistical reliability, with representative datasets selected for detailed interpretation. The electrochemical corrosion descriptors, specifically OCP and corrosion current density, were quantitatively determined by applying Tafel extrapolation to potentiodynamic polarization measurements [24]. Quality assurance procedures adhered to established electrochemical standards [25], incorporating rigorous surface preparation (sequential grinding to 2000-grit SiC paper), three-electrode calibration, and periodic instrument verification.

## 3. Results and Discussion

### 3.1. Phase Composition

**Figure 1:**
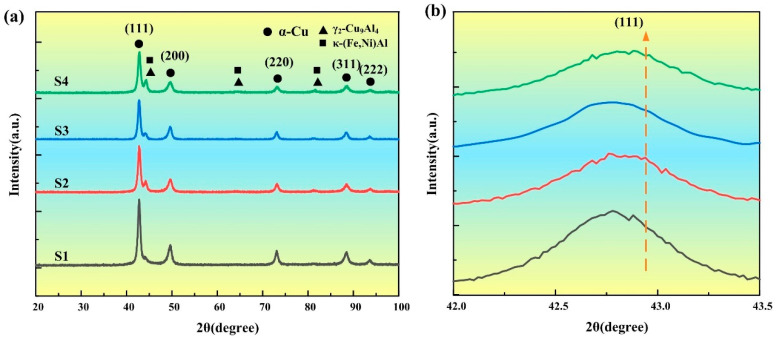
XRD patterns of as-CMTAM NAB deposited layers: (**a**) 20–100°; (**b**) partial enlarged image of (**a**).

As illustrated in Figure 1a, the X-ray diffraction profiles of specimens S1–S4 under varying heat input conditions display distinct diffraction peaks attributable to the face-centered cubic (FCC) α-phase solid solution, which was consistently identified throughout all fabricated layers, accompanied by a small amount of γ_2_-Cu_9_Al_4_ and κ phase (Fe,Ni)Al. The diffraction peaks of α-Cu correspond to the crystal planes (111), (200), (220), (311), and (222) in sequence. Figure 1b presents an enlarged view in the range of 42–43.5° near the main (111) FCC peak. Compared with the standard diffraction peak (PDF# 04-003-2430), the strongest diffraction peaks of each deposited layer corresponding to the (111) crystal plane have shifted to the left to varying degrees. According to Bragg’s law [26]:

(2) nλ=2d sin θ 

In the Bragg diffraction condition, the fundamental equation establishes geometric relationships between four critical parameters: (1) d, representing the interplanar spacing within the crystalline lattice structure; (2) θ, defined as the angle formed between the incident radiation path and the crystallographic diffraction plane; (3) n, an integer value corresponding to the diffraction order in constructive interference phenomena; and (4) λ, characterizing the wavelength of the incident electromagnetic radiation. A decrease in the spacing of the crystal zone leads to an increase in the diffraction angle θ. This phenomenon of the diffraction peak shifting to the left is consistent with the results of nickel element modified copper-based alloy coatings [27]. This phenomenon can be ascribed to the influence of thermal stress caused by the cold metal transition arc and the rapid solidification of the molten pool, which leads to lattice distortion. The lattice distortion effect enhances the strength and wear resistance of the deposited layer. In as-cast alloys, the β high-temperature stable phase can precipitate. However, under the cold metal transfer additive manufacturing process conditions, a eutectoid reaction occurs as described in Formula (3).

(3) $$\beta \overset{565\;{}^{\circ}C}{\to }\alpha +\gamma_{2}$$

This reaction is almost completely carried out, leaving no residual β phase or martensitic β’ phase. The γ_2_ is a solid solution of the Cu_9_Al_4_ (D83) cubic structure intermetallic compound, which imparts high microhardness and excellent toughness to the deposited layer formed via additive manufacturing [28]. As heat input increases, the cooling rate decreases, thereby promoting the occurrence of this eutectoid reaction. Solute atoms, including Al, Fe, and Ni, aggregate and precipitate from the matrix to form a dispersion-strengthening phase known as the κ phase. The κ phase exhibits distinct types, which can be categorized into iron-rich κ phases and nickel-rich κ phases based on their compositional characteristics. The clustering of soluble atoms such as Al, Fe, and Ni leads to their separation from the matrix and subsequent transformation into the κ phase, thereby contributing to dispersion strengthening and enhancing the mechanical properties of the alloy. Given the high hardness of the κ phase, its formation significantly improves the overall performance of the material. The formation mechanism of the κ phase has been investigated, where Tao et al. [4] demonstrated through thermodynamic calculations that the Gibbs free energy of the Ni-rich κ phase is negative, indicating its in situ formation capability. Similarly, Zhuang et al. [29] reported in their study of Fe_3_Al intermetallic compounds that the Gibbs free energy of the Fe-rich κ phase is also negative. The analysis was conducted based on the binary phase diagrams of the Fe-Al and Ni-Al systems [30,31], the melting points of iron-rich κ phases and nickel-rich κ phase are approximately 1540 °C and 1383 °C, respectively. Furthermore, the eutectoid reaction described in Formula (3) occurs at 565 °C. Consequently, the phase formation sequence for the nickel–aluminum bronze additive manufacturing alloy is κ-(Fe,Ni)Al → β → α-Cu + γ_2_, which aligns well with the findings from XRD analysis. The level of thermal input during processing significantly influences the distribution and morphology of the κ phase. Moreover, the κ phase that precipitates early serves as a heterogeneous nucleation site during solidification, playing a critical role in the evolution of the microstructure and the enhancement of mechanical properties.

### 3.2. Microstructure Analysis

**Figure 2:**
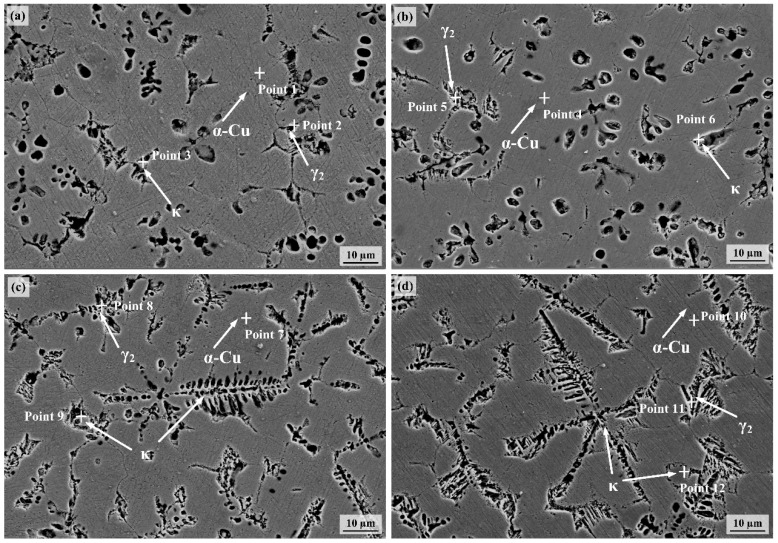
Microstructure of as-CMTAM NAB deposited layers: (**a**) 243.8 J/mm; (**b**) 378.8 J/mm; (**c**) 502.8 J/mm; (**d**) 644.7 J/mm.

The microstructure of CMTAM-NAB is illustrated in Figure 2. Energy dispersive spectroscopy (EDS) was employed to conduct quantitative scanning of elements in different regions of the microstructure, with the results summarized in Table 3. The microstructure primarily consists of α-Cu in the dendritic region and the interdendritic region. At a heat input of 243.8 J/mm (as shown in Figure 2a), the κ phase exhibits uniform distribution and small size, characterized by sub-micron features and retaining a certain net-shaped structure. This behavior can be attributed to the lower peak temperature of the molten pool heated by the cold metal transition arc and the faster cooling rate, which restricts the growth of intermetallic compounds. As the heat input increases to 378.8 J/mm (as shown in Figure 2b), the κ phase maintains relatively uniform distribution but shows slight coarsening in some areas. When the heat input further rises to 644.7 J/mm, as shown in Figure 2c,d, the κ phase significantly grows, expanding from sub-micron to micron or even tens of microns, and the net-shaped structure gradually transitions into a needle-shaped morphology. The κ phase precipitated under varying heat input conditions demonstrates differing degrees of coarsening. The reduction in cooling rate due to increased heat input promotes the formation and growth of intermetallic compounds. However, excessively high heat input may result in the inhomogeneous distribution characteristics of the κ phase. Admittedly, the κ phase is an aluminide phase containing Fe and Ni elements. These microstructural characteristics are representative of NAB after the CMTAM treatment. The abundant Fe, Ni, and Al atoms separated from the liquid metal rapidly diffuse in all directions, forming primary κ phase nuclei. With increasing heat input, the cooling rate decreases, prolonging solidification time. Larger temperature fluctuations, energy fluctuations, and compositional variations contribute to the gradual increase in the number of κ phase nuclei and the growth of the κ phase.

In summary, under different heat input conditions, the primary distinctions among alloys are manifested in the relative phase content, phase size, and the shape as well as distribution characteristics of phases within the microstructure. With the increase in heat input, the κ phase gradually undergoes coarsening. According to the analysis of the equilibrium phase characteristics of NAB alloys, the microhardness of the α phase, γ_2_ phase and κ phase are 200–270 HV, 360–570 HV and greater than 700 HV, respectively [32]. Among them, the fine and dispersed κ phase is of great significance for achieving high microhardness and excellent wear resistance in nickel–aluminum bronze alloys.

### 3.3. Electron Backscatter Diffraction Analysis

**Figure 3:**
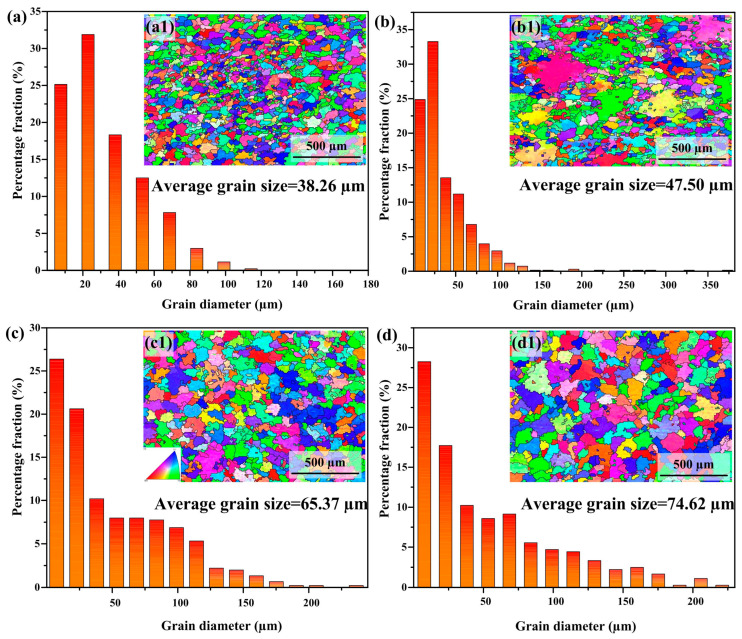
IPF map on XY direction and histogram of grain size distribution of as-CMTAM NAB deposited layers: (**a1**,**a**) 243.8 J/mm; (**b1**,**b**) 378.8 J/mm; (**c1**,**c**) 502.8 J/mm; (**d1**,**d**) 644.7 J/mm.

The inverse pole figure (IPF) and average grain size of the nickel–aluminum bronze deposited layer were characterized using EBSD. The XY plane was selected as the measurement direction, and the results are presented in Figure 3. Specifically, Figure 3(a1–d1) correspond to heat input (HI) values of 243.8, 378.8, 502.8, and 644.7 J/mm, respectively. Due to the random orientation of grains in all four deposited layers, no texture formation was observed. This randomness in grain orientation during solidification and growth can be attributed to the continuous melting and resolidification processes occurring during CMTAM, which effectively hindered the development of any preferred texture. To investigate the effect of HI on grain size, the grain size distribution histograms for the deposited layers are shown in Figure 3(a–d). The average grain sizes of the deposited layers corresponding to thermal inputs of 243.8, 378.8, 502.8, and 644.7 J/mm were measured as 38.26 μm, 47.50 μm, 65.37 μm, and 74.62 μm, respectively. Notably, a significant increase in grain size with increasing thermal input is evident.

**Figure 4:**
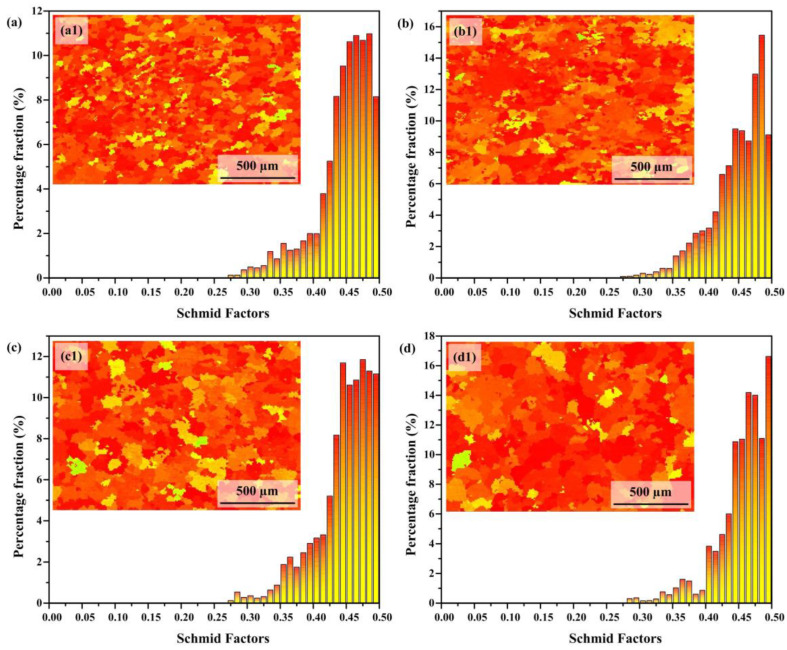
Schmid factors maps and corresponding Schmid factors distribution histograms: (**a1**,**a**) 243.8 J/mm; (**b1**,**b**) 378.8 J/mm; (**c1**,**c**) 502.8 J/mm; (**d1**,**d**) 644.7 J/mm.

As shown in Figure 4, the Schmidt factors of the nickel–aluminum bronze additive manufacturing deposition layers are 0.41, 0.44, 0.45, and 0.47, respectively. Specifically, samples S1 and S2 exhibit a certain degree of distribution below the Schmitt factor value of 0.4; however, as heat input (HI) increases, the Schmitt factor becomes increasingly concentrated in the higher range, with a marked reduction in its distribution below 0.4. For face-centered cubic (FCC) metals, the primary slip system is {111}<110>. Based on the critical shear stress law governing single-crystal metal deformation [33], the following formula can be derived:

(4) $$\sigma_{0.2}=\frac{\tau_{C}}{{\left(\cos\varphi \cos\lambda \right)}_{max}}$$

In the constitutive model, σ_0.2_ corresponds to the 0.2% offset yield strength; τ_C_ signifies the material-dependent critical shear stress; φ designates the angular orientation between slip plane normal and loading axis; while λ characterizes the angular deviation between slip direction and principal stress vector. Here, (cos φ cos λ)_max_ corresponds to the maximum value of the Schmitt factor. It is evident that a larger Schmitt factor demonstrates a greater propensity for grains to undergo plastic deformation, thereby exhibiting reduced resistance to deformation. Consequently, it can be inferred that excessive heat input may result in a decrease in the strength of the NAB additive manufacturing deposition layer.

### 3.4. Microhardness

**Figure 5:**
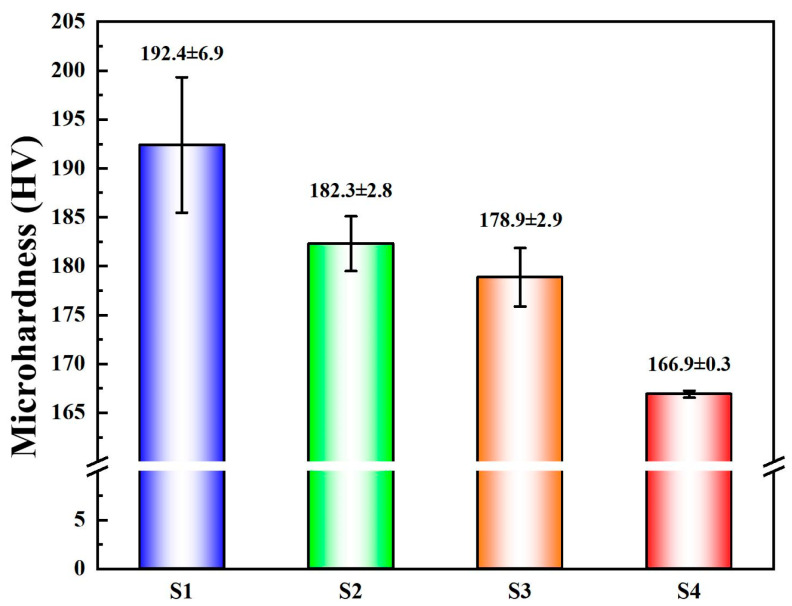
The average microhardness of S1, S2, S3, and S4.

Figure 5 presents the surface microhardness of the deposition layers. The measured surface microhardness values for these four samples are 192.4 ± 6.9 HV, 182.3 ± 2.8 HV, 178.9 ± 2.9 HV, and 166.9 ± 0.3 HV, respectively, indicating a decreasing trend in microhardness. Wang et al. [34] also reached a similar conclusion when preparing WC-Co/X32 steel joints by using the RSW method: as the current increased, the microhardness decreased. This phenomenon can be attributed to two primary factors: grain size variation and the influence of κ-phase precipitation strengthening.

Firstly, under low heat input (HI) conditions, fine grains form, resulting in the highest microhardness observed in the S1 sample. As heat input increases, the grains in the nickel–aluminum bronze additive manufacturing deposition layers gradually coarsen, leading to a reduction in microhardness. From the variation trend of the Schmidt factor in Figure 4, it can be observed that as the input quantity increases, the alloy gradually shows a softening tendency. According to the classical Hall–Petch relationship theory [35], the correlation between microhardness and grain size for coarse-grained materials can be expressed as follows:

(5) $$H=H_{0}+\frac{K_{y}}{\sqrt{\varphi }}$$

In this formulation, H denotes the microhardness; φ corresponds to the mean grain diameter; H_0_ signifies the frictional hardness unaffected by grain dimensions; and K_y_ represents the characteristic Hall–Petch coefficient. From Equation (5), it is evident that microhardness exhibits an inverse relationship in proportion to the square root of the average grain diameter, indicating a decrease as the grain size increases. Therefore, grain coarsening significantly reduces microhardness. Secondly, the κ-phase precipitation-strengthening effect predominantly governs the microhardness variations. Extensive research has substantiated that κ-phase precipitation strengthening constitutes the predominant contributor to the superior mechanical properties exhibited by nickel-aluminum bronze alloys [36,37]. Hasan et al. [38] found that the κ phase exhibits a K–S orientation relationship with the α-Cu matrix, characterized by high crystallographic matching and low interfacial energy, ensuring stable bonding between the precipitated phase and the matrix. Under low heat input conditions, fine and uniformly dispersed κ phases are distributed throughout the matrix, forming numerous strengthening particles. These particles effectively pin dislocations at the grain boundaries, thereby significantly enhancing material hardness and strength. However, as heat input increases, the κ phase grows larger, reducing its volume fraction per unit area and weakening the strengthening effect. Additionally, the uneven distribution of larger κ-phase particles may induce stress concentration, further diminishing the strengthening effect and potentially decreasing the hardness and strength of the deposition layer. In summary, the decreasing trend in microhardness with increasing heat input is primarily attributed to grain coarsening and the coarsening and uneven distribution of the κ phase.

### 3.5. Corrosion Behavior

**Figure 6:**
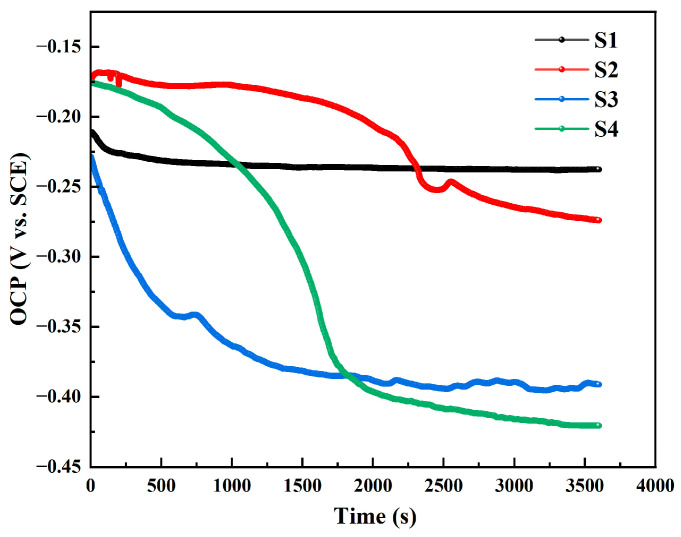
Evolution of OCP for the as-CMTAM NAB deposited layers immersed in a 3.5% NaCl solution.

Figure 6 shows the variation in open circuit potential (OCP) over time for CMTAM nickel–aluminum bronze samples under different heat input conditions. It can be seen that for sample 1, the OCP drops rapidly only within the first 150 s of immersion and then gradually becomes more negative at a slower rate. For samples 2 to 4, the OCP drops rapidly within the first 2000 s of immersion and then gradually stabilizes, becoming more negative at a slower pace. This behavior may be attributed to the corrosive effect of chloride ions in seawater. The corrosion potentials (E_corr_) of samples 1 to 4 after 1 h of immersion are approximately −237 mV, −274 mV, −391 mV, and −421 mV, respectively.

**Figure 7:**
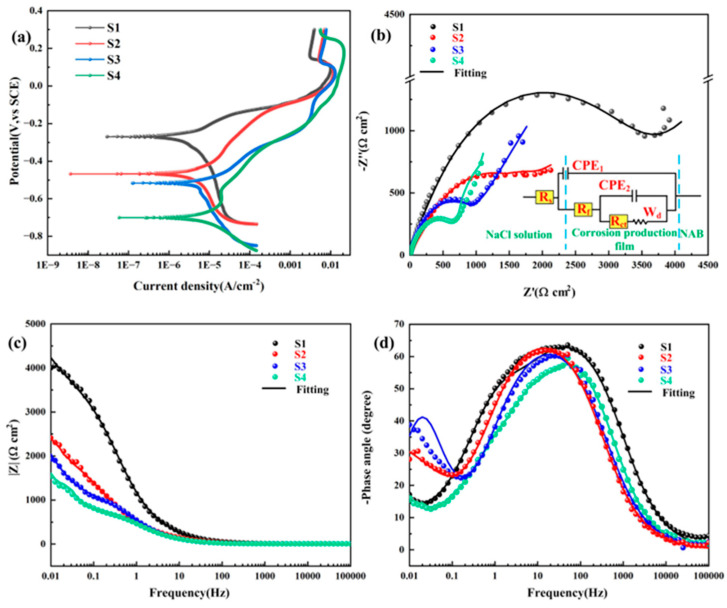
Corrosion characteristics: (**a**) Potentiodynamic polarization curves; (**b**) Nyquist diagram; Bode plots: (**c**) Bode plots of |Z| vs. frequency and (**d**) Bode plots of degree vs. frequency.

Figure 7 illustrates the relationship between the potentiodynamic polarization curves of as-CMTAM nickel–aluminum bronze in a 3.5 wt% NaCl solution and heat input. Through analysis of these curves, key polarization parameters, including the potential obtained from the cathodic direction of the polarization curve (E) and corrosion current density (i_corr_) were experimentally measured through electrochemical analysis and systematically compiled in Table 4. The results demonstrate that the polarization curves of the four samples exhibit similar profiles and a tendency to overlap in the anodic polarization region, suggesting consistent dissolution and oxidation kinetics. This can be attributed to the electrochemical behavior of nickel–aluminum bronze being predominantly governed by its primary copper element [39]. As input increases, the corrosion potential decreases from −269.61 ± 40 mV to −700.94 ± 15 mV, indicating a significant shift towards more negative values. Simultaneously, the corrosion current density rises from 2.56 ± 0.04 µA/cm^2^ to 7.52 ± 0.07 µA/cm^2^, reflecting a reduction in corrosion resistance of the materials.

**Figure 8:**
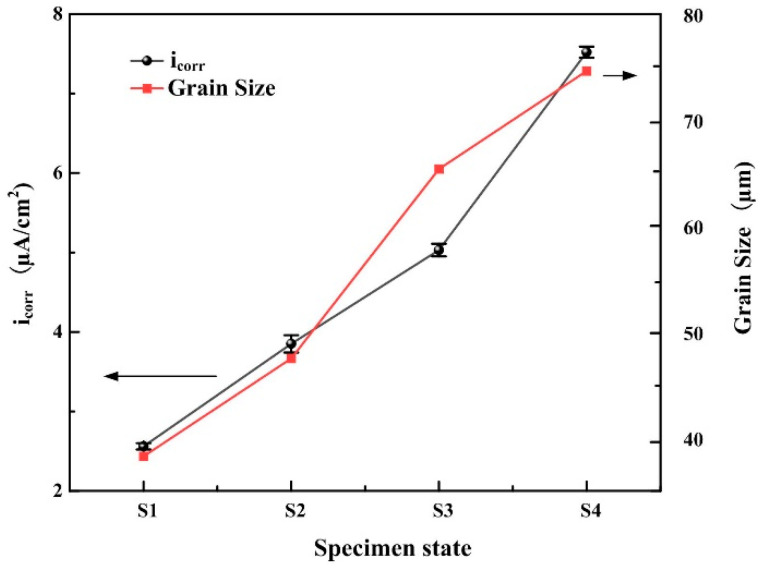
Variation in i_corr_ and grain size as a function of heat input.

Figure 8 further clarifies the variation trends of i_corr_ and grain size (derived from the statistics of average grain size through EBSD in Figure 3) with heat input during the additive manufacturing process. Along the increasing heat input path (S1 to S4), the two parameters of i_corr_ and grain size demonstrate consistent unidirectional variation trends. The extent of surface dissolution or passivation is closely associated with total grain boundary length, which serves as an indicator of overall surface reactivity. Grain boundaries act as physical barriers that effectively inhibit corrosion propagation [40,41]. With progressive elevation of thermal energy input, grain coarsening induces depressed charge carrier mobility at boundary interfaces, consequently limiting available nucleation sites for surface reaction products in metallic substrates [42,43]. Furthermore, a lower grain boundary density weakens repassivation kinetics and reduces the electronic work function [44], which is detrimental to the formation of a stable passivation film. The promoting effect of the pinning mechanism on mechanical adhesion between the passivation film and the metal substrate also diminishes gradually, ultimately leading to a weaker surface film strength [45,46]. It should be noted that the aforementioned phenomena cannot be entirely attributed to variations in grain size.

The corrosion behavior of materials is closely associated with their phase composition and distribution. After the CMTAM treatment, nickel–aluminum bronze primarily consists of α-Cu, γ_2_, and κ phases. Among these, the γ_2_ phase exhibits relatively poor corrosion resistance [47], with a lower potential compared to the α-Cu phase, whereas the κ phase demonstrates a higher potential. Consequently, a significant potential difference arises between the different constituent phases. During this process, the κ phase functions as the cathode, while the Cu-rich γ_2_ phase and α phase serve as the anodes, leading to selective phase corrosion. Figure 7b–d display the Nyquist and Bode plots of the samples in a 3.5 wt% NaCl solution. The impedance spectra reveal a prominent semi-circular capacitive loop at high frequencies, indicative of a single time constant within this frequency range, reflecting the charge transfer process. At low frequencies, a Warburg line is observed, which corresponds to diffusion phenomena. The measured impedance data were fitted using ZsimpWin software, and the associated equivalent circuit configuration is schematically represented in the annotated inset of Figure 7b. The derived electrochemical impedance parameters are systematically tabulated in Table 5. Here, in the equivalent circuit model, R_s_ quantifies the ohmic resistance of the electrolyte solution; R_f_ quantifies the film resistance, whereas R_ct_ characterizes the interfacial electron-transfer resistance. Considering the capacitance dispersion caused by surface roughness and heterogeneities, a Constant Phase Element (CPE) is introduced to characterize the non-ideal capacitor [48] and achieve optimal fitting. Therefore, CPE_1_ and CPE_2_ in Figure 7b are associated with the non-ideal capacitance of the passive film and the double-charge layer, respectively. The CPE impedance can be mathematically described through the subsequent equation [49,50,51]:

(6) $$Z_{\mathrm{CPE}}={\left[Q\left(j\omega \right)n\right]}^{-1}$$

In this notation, Q denotes the constant phase element (CPE) magnitude; j corresponds to the imaginary unit; ω indicates the angular frequency; and n represents the CPE power coefficient. The symbol W_d_ characterizes the Warburg diffusion impedance associated with mass transport between the NAB surface and the NaCl electrolyte [52,53,54]. Its physical meaning can be summarized as the solution resistance R_s_ is in series with a parallel structure composed of the constant phase element CPE_1_ (characterizing the non-ideal capacitance of the metal surface film, where n1 reflects its uniformity) and the sub-circuit R_f_ (charge transfer resistance). The inner layer further nests a series structure of CPE_2_ (the double-layer capacitance at the metal interface) and R_ct_-W (R_ct_ is the charge transfer resistance of the corrosion reaction, and W represents the diffusion limitation in the corrosion product film or the electrolyte). The overall model analyzes the coupling mechanism of alloy failure, interface reaction and mass transfer process through multiple time constants. The circuit featuring two parallel time constants (Figure 7a) has been employed to account for both passivity behavior (CPE_1_, R_f_) and corrosion activity (CPE_2_, R_ct_). The simulation quality was assessed using the chi-square value (χ^2^), which ranged from 3.88 × 10⁻^4^ to 3.76 × 10⁻^3^, indicating a high degree of agreement between the measured and calculated data [55,56,57,58]. As heat input increases, the diameter of the semicircle in the Nyquist plot decreases significantly, a trend also evident in the Bode plot: at 0.01 Hz, the absolute impedance value diminishes with increasing heat input. Notably, sample S4 demonstrates significant decreases in both the Nyquist semicircle diameter and the 0.01 Hz impedance modulus observed in Bode plots, indicative of substantial corrosive degradation. Within the frequency range of 10 mHz to 100 kHz, changes in the impedance curve are independent of frequency, and the high-frequency phase angle approaches 0°. The reduction in the diameter of the capacitive loop and the charge transfer resistance for samples S3 and S4 indicates that the material’s corrosion resistance decreases with increasing heat input, consistent with the Tafel polarization data. These phenomena can be attributed to the intensification of galvanic corrosion caused by different phases forming in a conductive environment under high heat input conditions, further promoting selective phase corrosion.

Electrochemical corrosion processes in cold metal transfer additive manufactured Cu–Al alloy systems is characterized by anodic dissolution occurring at the Cu-rich phase, whereas the cathodic reaction proceeds via predominant oxygen reduction, as depicted in the following equation [59]:

(7) $$\mathrm{Cu}\mathrm{Cl}+{\mathrm{Cl}}^{-}\;\to \;\mathrm{Cu}{\mathrm{Cl}}_{2}^{-}$$

(8) $$O_{2}+2H_{2}O+4e^{-}\;\to \;4OH^{-}$$

(9) $$2\mathrm{CuC}l_{2}^{-}+2OH^{-}\;\to \;Cu_{2}O+H_{2}O+4Cl^{-}$$

A stratified oxide architecture develops through selective oxidation, with cuprous oxide (Cu_2_O) passivation layers evolving in the surface region concomitant with aluminum oxide (Al_2_O_3_) inner region formation within the subsurface zone, as expressed below [60]:

(10) $$\mathrm{Al}+4Cl^{-}\;\to \;\mathrm{AlC}l_{4}^{-}+3e^{-}$$

(11) $$2\mathrm{AlC}l_{4}^{-}+3H_{2}O\;\to \;Al_{2}O_{3}+6H^{+}+8Cl^{-}$$

Under lower heat input conditions, the alloy undergoes an extremely high cooling rate. The restricted diffusion of solute atoms in both solid and liquid states leads to relatively fine precipitated phases formed by the eutectoid reaction, as observed in samples S1 and S2 (Figure 2a,b). In these samples, the distribution of Al elements is more uniform, resulting in the formation of a more continuous Al_2_O_3_ protective film. Conversely, with increasing heat input, as demonstrated in samples S3 and S4 (Figure 2c,d), the precipitated phases significantly coarsen, and the distribution of Al elements exhibits segregation. This non-uniform solute distribution may considerably affect the protective performance of the aforementioned surface oxide film.

## 4. Conclusions

Nickel–aluminum bronze deposits with varying HI were produced via CMTAM technology, followed by comprehensive characterization of their microstructural features, hardness properties, and electrochemical corrosion behavior. The key findings are summarized as follows:

(1) The NAB deposit layer is mainly composed of α-Cu, accompanied by a small amount of γ_2_-Cu_9_Al_4_ and κ phase, with varying degrees of lattice distortion. As the heat input gradually increases, the κ phase gradually coarsens.
(2) As the heat input increases, both the crystalline domain dimensions and the crystallographic orientation parameter exhibit a proportional rise. The average grain size increases from 38.26 μm to 74.62 μm. The Schmidt factor increases from 0.41 to 0.47. The alloy phase gradually softens. When the heat input is 243.8 J/mm, the grain size is the smallest and the value of the Schmidt factor is the smallest.
(3) The variation in grain size and the κ-phase precipitation strengthening mechanism exert a governing effect on the microhardness evolution within the as-deposited layers. The average microhardness decreased from 192.4 ± 6.9 HV to 166.9 ± 0.3 HV with the increase in input. The coarsening of grains and κ phase, as well as their uneven distribution, significantly reduced the microhardness.
(4) With the increase in heat input, the corrosion potential decreased significantly from −269.61 ± 40 mV to −700.94 ± 15 mV, while the corrosion current density rose markedly from 2.56 ± 0.04 µA/cm^2^ to 7.52 ± 0.07 µA/cm^2^. The deterioration of corrosion-resistant properties is predominantly governed by two predominant mechanisms: (1) grain coarsening; and (2) the coarsening of precipitated phases, which induces Al segregation and consequently degrades the protective performance of the Al_2_O_3_ film.

Although this paper has verified the microhardness and corrosion resistance of CMTAM nickel–aluminum bronze material under simulated working conditions, its large-scale industrial application still needs to address the challenge of establishing a life prediction model based on actual service data. Subsequent research will focus on the above issues.

## Figures and Tables

**Table 1:** Nominal chemical composition of the substrate and welding wires (wt%).

	Cu	Al	Fe	Ni	Mn	Pb	Si	Zn
ERCuNiAl	Bal.	8.73	5.9	4.52	1.39	0.005	0.058	0.002
Substrate	Bal.	8.62	2.32	0.005	0.258	0.027	0.149	0.021

**Table 2:** Process parameters used during CMTAM of nickel–aluminum bronze.

Samples	Current (A)	Voltage (V)	WFS (m/min)	TS (mm/min)	Stick Out (mm)	HI (J/mm)
S1	137	12.7	6	480	14	243.8
S2	158	15.0	7			378.8
S3	184	17.1	8			502.8
S4	209	19.3	9			644.7

**Table 3:** Chemical composition in different regions as marked in Figure 2 (wt%).

Sample	Point	Cu	Al	Ni	Fe	Mn	Phases Identified
S_1_	1	85.2	5.7	3.9	3.8	1.4	α-Cu
	2	78.1	8.5	4.9	6.8	1.7	γ_2_
	3	10.2	18.8	28.7	41.1	1.2	κ
S_2_	4	84.5	5.2	3.7	5.3	1.3	α-Cu
	5	78.8	8.8	4.4	6.9	1.1	γ_2_
	6	8.5	17.9	31.1	40.7	1.8	κ
S_3_	7	83.1	6.1	3.9	5.6	1.3	α-Cu
	8	77.8	11.3	4.7	6.2	1.9	γ_2_
	9	20.6	18.2	26.8	33.4	1.0	κ
S_4_	10	81.8	5.2	4.9	6.6	1.5	α-Cu
	11	73.3	9.3	8.1	7.3	2.0	γ_2_
	12	6.8	16.8	27.5	48.2	0.7	κ

**Table 4:** Electrochemical parameters of as-CMTAM nickel–aluminum bronze alloys in 3.5 wt% NaCl solution.

Specimens	E (mV)	i_corr_ (µA/cm^2^)
S1	−269.61 ± 40	2.56 ± 0.04
S2	−466.62 ± 10	3.85 ± 0.11
S3	−517.77 ± 20	5.03 ± 0.08
S4	−700.94 ± 15	7.52 ± 0.07

**Table 5:** The fitting results of EIS data for as-CMTAM nickel–aluminum bronze alloys in 3.5 wt% NaCl solution.

Gradient	R_s_ (Ω·cm^2^)	Q_f_ (µF·cm^−2^·s^n−1^)	n_1_	R_f_ (Ω·cm^2^)	Q_ct_ (µF·cm^−2^·s^n−1^)	n_2_	R_ct_ (Ω·cm^2^)	W_d_ (S·cm^−2^·s^1/2^)	Chi-Squared
S1	8.149	11.700 × 10^−5^	0.80	1152	1.067 × 10^−4^	0.80	2485	34.60 × 10^−4^	8.21 × 10^−4^
S2	7.702	1. 651 × 10^−4^	0.82	1047	4.893 × 10^−4^	0.63	1966	99.55 × 10^−4^	5.36 × 10^−4^
S2	7.714	3. 141 × 10^−4^	0.79	947	5.076 × 10^−3^	0.85	1284	27.82 × 10^−4^	5.41 × 10^−4^
S4	7.102	3.254 × 10^−4^	0.78	185.8	5.941 × 10^−4^	0.80	707	10.10 × 10^−4^	4.83 × 10^−3^

## Data Availability

The original contributions presented in this study are included in the article. Further inquiries can be directed to the corresponding authors.

## References

[1] Chen W., Chen Y.H., Zhang T.M., Wen T.T., Feng X.S., Yin L.M. (2020). Effects of Location on the Microstructure and Mechanical Properties of Cu-8Al-2Ni-2Fe-2Mn Alloy Produced Through Wire Arc Additive Manufacturing. J. Mater. Eng. Perform..

[2] Li Y., Lian Y., Sun Y.J. (2019). Cavitation Erosion Behavior of Friction Stir Processed Nickel Aluminum Bronze. J. Alloys Compd..

[3] Cai X., Chang S.K., Yang M.M., Li S.J., Wang X., Qiao Y.X., Zhou J., Xue F. (2025). From cast to wire-arc DED: An investigation on NAB alloy MIC resistance. Surf. Coat. Technol..

[4] Rosalbino F., Carlini R., Soggia F., Zanicchi G., Scavino G. (2012). Influence of rare earth metals addition on the corrosion behaviour of copper in alkaline environment. Corros. Sci..

[5] Ding Y., Zhao R., Qin Z.B., Wu Z., Wang L.Q., Liu L., Lu W.J. (2019). Evolution of the Corrosion Product Film on Nickel-Aluminum Bronze and Its Corrosion Behavior in 3.5 wt % NaCl Solution. Materials.

[6] Behbahani K.M., Rayner A.J., Bishop D.P., Nasiri A. (2025). Impact of fabrication method on the corrosion behavior of heat-treated nickel aluminum bronze (NAB) alloy: A comparison of laser powder bed fusion (L-PBF) and laser directed energy deposition (L-DED) techniques. Int. J. Electrochem. Sci..

[7] Alam S., Marshall R.I., Sasaki S. (1996). Metallurgical and tribological investigations of aluminum bronze bushes made by a novel centrifugal casting technique. Tribol. Int..

[8] Feldshtein E.E., Devojno O., Kardapolava M., Lutsko N., Patalas-Maliszewska J. (2021). On the Features of Composite Coating, Based on Nickel Alloy and Aluminum-Iron Bronze, Processed by Direct Metal Deposition. Materials.

[9] Cong B., Ouyang R., Qi B., Ding J.L. (2016). Influence of cold metal transfer process and its heat input on weld bead geometry and porosity of aluminum-copper alloy welds. Rare Met. Mater. Eng..

[10] Müller V., Fasselt J.M., Klötzer-Freese C., Kruse T., Kleba-Ehrhardt R., Biegler M., Rethmeier M. (2025). Recycling Nickel Aluminium Bronze Grinding Chips to Feedstock for Directed Energy Deposition via Impact Whirl Milling: Investigation on Processability, Microstructure and Mechanical Properties. Addit. Manuf..

[11] Yang J., Wu F., Bai B., Wang G.S., Yang L., Zhou S.F., Lei J.B. (2020). Effect of Cr additions on the microstructure and corrosion resistance of Diode laser clad CuAl10 coating. Surf. Coat. Technol..

[12] Dana M., Zetkov I., Hanzl P. (2019). The influence of a ceramic recoater blade on 3D printing using direct metal laser sintering. Manuf. Tech..

[13] Utyaganova V., Vorontsov A., Gurianov D., Shamarin N., Chumaevskii A., Rubtsov K., Savchenko N., Tarasov S. (2023). Effect of Mg admixing on strength and corrosion of electron beam additive manufactured AlSi1_2_ on the AA5056 substrate. Mater. Charact..

[14] Lv J.M., Alexandrov L.V., Luo K.Y., Lu H.F., Lu J.Z. (2022). Microstructural evolution and anisotropic regulation in tensile property of cold metal transfer additive manufactured Ti6Al4V alloys via ultrasonic impact treatment. Mater. Sci. Eng. A.

[15] Gou J., Wang Z.J., Hu S.S., Shen J.Q., Tian Y.B., Zhao G.C., Chen Y.Q. (2020). Effects of trace Nb addition on microstructure and properties of Ti–6Al–4V thin-wall structure prepared via cold metal transfer additive manufacturing. J. Alloys Compd..

[16] Chen Z.W., Liang Y.N., Xu C., Zhang X.Y., Fan J.K., Liu J., Kong J., Wang K.H., Peng Y. (2024). Improving the product of strength and ductility and corrosion resistance by adding He shielding gas in the CMT additive manufacturing process of Ti6Al4V. J. Alloys Compd..

[17] Dharmendra C., Hadadzadeh A., Amirkhiz B.S., Janaki Ram G.D., Mohammadi M. (2019). Microstructural evolution and mechanical behavior of nickel aluminum bronze Cu-9Al-4Fe-4Ni-1Mn fabricated through wire-arc additive manufacturing. Addit. Manuf..

[18] Aliyu A., Bishop D.P., Nasiri A. (2025). Effect of heat treatment on microstructural evolution and corrosion behavior of wirearc additive manufactured nickel aluminum bronze alloy. Corros. Sci..

[19] Shen C., Pan Z.X., Ding D.H., Yuan L., Nie N., Wang Y., Luo D.Z., Cuiuri D., Duin S.V., Li H.J. (2018). The influence of post-production heat treatment on the multi-directional properties of nickel-aluminum bronze alloy fabricated using wire-arc additive manufacturing process. Addit. Manuf..

[20] Peng Y.H., Zhao L.J., Cui X.Y., Xiong T.Y., Wang J.Q. (2024). Effect of heat treatment on the gas-atomized nickel-aluminum bronze feedstock powders for cold spray. Surf. Coat. Technol..

[21] Wang D.C., Tao X.P., Wang X.G., Zhang R., Zhou Z.J., Zhang C.H., Zhang S., Wu C.L., Sun X.F., Zhou Y.Z. (2025). Normalizing temperature dependence on microstructure, mechanical and wear properties of a novel high-vanadium high-speed steel. Mater. Charact..

[22] Li T.C., Zhang S.P., Xie M., Song X.L., Lei J.B. (2024). Directed energy deposition of aluminium bronze/GNPs composites: Microstructural evolution for enhanced wear resistance. Tribol. Int..

[23] Wu H., Wang Z.Y., Wang M.S., Wang R., Zhang S., Zhang C.H., Wu C.L., Chen H.T., Chen J. (2025). Microstructure evolution, corrosion and corrosive wear properties of NbC-reinforced FeNiCoCr-based high entropy alloys coatings fabricated by laser cladding. Eng. Fail. Anal..

[24] Marginean G., Utu D. (2010). Microstructure refinement and alloying of WC–CoCr coatings by electron beam treatment. Surf. Coat. Technol..

[25] Zhang R., Tao X.P., Wang X.G., Zhang R., Zhang C.H., Zhang S., Wu C.L., Sun X.F., Zhou Y.Z., Cui C.Y. (2025). Effect of tempering temperature on the microstructure, mechanical and wear failure behaviours of a novel high vanadium high-speed steel. Steel Res. Int..

[26] Yang Q., Zhao L.R. (2008). Characterization of nano-layered multilayer coatings using modified Bragg law. Mater. Charact..

[27] Huang S.W., Zhou P.F., Luo F.X., Chen H.M., Xie W.B., Zhang W.J., Wang H., Yang B., Peng B.F. (2023). Effects of Ni and Mn contents on precipitation and strengthening behavior in Cu-Ni-Mn ternary alloys. Mater. Charact..

[28] Svirid A.E., Pushin V.G., Kuranova N.N., Luk’yanov A.V., Pushin A., Uksusnikov A.N., Ustyugov Y.M. (2017). The structure–phase transformations and mechanical properties of the shape memory effect alloys based on the system Cu-Al-Ni. Mater. Today Proc..

[29] Zhuang L.Z., Buekenhout L., Duszczyk J. (1994). Reaction phase-forming and mechanical properties of Fe_3_Al produced from elemental powders. Scr. Met. Mater..

[30] Wen J., Cui H., Wei N., Song X., Zhang G., Wang C., Song Q. (2017). Effect of phase composition and microstructure on the corrosion resistance of Ni-Al intermetallic compounds. J. Alloys Compd..

[31] Zheng K.X., Yu D.T., Liu J.L., Wu C.L., Zhang S., Zhang C.H., Wang Q., Zhang D. (2025). Laser cladding of FeCoCrNiTi high-entropy alloy coatings to modulate the microstructure and enhance the tribo-corrosion behavior on 304 stainless steel. Surf. Coat. Technol..

[32] Zhao T.Y., Wang M.S., Wu H., Xu T.Z., Wu C.L., Zhang C.H., Zhang S., Chen H.T., Chen J. (2025). Effect of aging treatment on cavitation erosion resistance and slurry erosion resistance of laser directed energy deposition martensitic aging steel. Mater. Today Commun..

[33] Jeng Y.R., Tsai P.C., Chiang S.H. (2013). Effects of grain size and orientation on mechanical and tribological characterizations of nanocrystalline nickel films. Wear.

[34] Wang R., Zhang S., Hu Y., Meng Q.H., Fu B., Zhao Y.W., Wu C.L., Li G.M., Qin J., Wang J.P. (2025). Effects of welding current on microstructure evolution and mechanical behavior of WC-Co/X32 steel joints by resistance spot welding. Int. J. Refract. Met. Hard Mater..

[35] Varghese P., Vetrivendan E., Dash M.K., Ningshen S., Kamaraj M., Mudali U.K. (2019). Weld overlay coating of Inconel 617 M on type 316 L stainless steel by cold metal transfer process. Surf. Coat. Technol..

[36] Aliyu A., Bishop D.P., Nasiri A. (2024). Effect of heat input on bead geometry and mechanical properties in wire arc additive manufacturing of a nickel aluminum bronze alloy. J. Mater. Res. Technol..

[37] Cai X., Yang M.M., Qiao Y.X., Wang Z., Zhou J., Xue F.X. (2023). Experimental investigation on wear resistance and corrosion behavior of nickel-aluminum bronze alloy fabricated by wire-arc additive manufacturing. J. Mater. Res. Technol..

[38] Hasan F., Jahanafrooz A., Lorimer G.W., Ridley N. (1982). The Morphology, Crystallography, and Chemistry of Phases in As-Cast Nickel-Aluminum Bronze. Metall. Trans. A.

[39] Wu Z., Cheng Y.F., Liu L., Lv W., Hu W. (2015). Effect of heat treatment on microstructure evolution and erosion–corrosion behavior of a nickel–aluminum bronze alloy in chloride solution. Corros. Sci..

[40] Aung N.N., Zhou W. (2010). Effect of grain size and twins on corrosion behaviour of AZ31B magnesium alloy. Corros. Sci..

[41] Gao M., Jia C., Ni D., Luan Y., Ma Z.J. (2022). Influence of microstructure modification on corrosion resistance of friction stir processing biodegradable Mg-Zn-Nd alloy. Mater. Technol..

[42] Li J., Lu Y.H., Tu X.H., Li W. (2018). The effects of subsurface microstructure evolution on fretting wear resistance of nickel-based alloy. Wear.

[43] Culpan E.A., Rose G. (1978). Microstructural characterization of cast Nickel Aluminum bronze. J. Mater. Sci..

[44] Wang X.Y., Li D.Y. (2002). Mechanical and electrochemical behavior of nanocrystalline surface of 304 stainless steel. Electrochim. Acta.

[45] Tao S., Li D.Y. (2006). Tribological, mechanical and electrochemical properties of nanocrystalline copper deposits produced by pulse electrodeposition. Nanotechnology.

[46] Elkedim O., Cao H.S., Meunier C., Gaffet E. (1998). Preparation of nanocrystalline copper by hot and cold compaction: Characterization of mechanical and electrochemical properties. Mater. Sci. Forum..

[47] Zhai W.Z., Zhao Y.J., Zhou R.H., Lu W.L., Zhai W.C., Liu X.J., Zhou L.P., Chang S.P. (2022). Additively manufactured (Fe, Ni)Al-reinforced nickel aluminum bronze with nearly-isotropic mechanical properties in build and transverse directions. Mater. Charact..

[48] Jorcin J.B., Orazem M.E., Pébère N., Tribollet B. (2006). CPE analysis by local electrochemical impedance spectroscopy. Electrochim. Acta.

[49] El-Sherif R.M., Ismail K.M., Badawy W.A. (2004). Effect of Zn and Pb as alloying elements on the electrochemical behavior of brass in NaCl solutions. Electrochim. Acta.

[50] Qin Z.B., Luo Q., Zhang Q., Wu Z., Liu L., Shen B., Hu W.B. (2018). Improving corrosion resistance of nickel-aluminum bronzes by surface modification with chromium ion implantation. Surf. Coat. Technol..

[51] Qin Z.B., Xia D.H., Zhang Y.W., Wu Z., Liu L., Lv Y.T., Liu Y.C., Hu W.B. (2020). Microstructure modification and improving corrosion resistance of laser surface quenched nickel–aluminum bronze alloy. Corros. Sci..

[52] Wang T., Wang Z.Y., Wang R., Wang M.S., Wu C.L., Zhang C.H., Zhang S., Chen H.T., Chen J. (2025). Effect of solution aging treatment on corrosion resistance and erosion resistance of laser metal deposition 17-4PHss. Eng. Fail. Anal..

[53] Qin Z.B., Zhang Q., Luo Q., Wu Z., Shen B., Liu L., Hu W.B. (2018). Microstructure design to improve the corrosion and cavitation corrosion resistance of a nickel-aluminum bronze. Corros. Sci..

[54] Siemek K., Eseev M.K., Horodek P., Kobets A.G., Kuziv I.V. (2021). Defects studies of nickel aluminum bronze subjected to cavitation. Appl. Surf. Sci..

[55] Zhang H.F., Wu H., Wang Z.Y., Wang R., Zhang S., Zhang C.H., Wu C.L., Chen J., Chen H.T. (2025). In-situ synthesized (Ti,Nb)C reinforced austenitic stainless steel by laser cladding: New insights into microstructure evolution and corrosion behavior. Mater. Today Commun..

[56] Yang F.F., Kang H.J., Guo E.Y., Li R.G., Chen Z.N., Zeng Y.H., Wang T.M. (2018). The role of nickel in mechanical performance and corrosion behaviour of nickel-aluminium bronze in 3.5 wt.% NaCl solution. Corros. Sci..

[57] Wu H., Wang M.S., Zhang S., Wang R., Zhang C.H., Wu C.L., Chen H.T., Chen J. (2025). Cavitation erosion characteristics and dominant factors of a novel FeNiCoCrMo0.3Nb0.5 hypoeutectic high entropy alloys coating. Wear.

[58] Song S.L., Li D.G., Chen D.R., Liang P. (2022). The role of Ti in cavitation erosion and corrosion behaviours of NAB alloy in 3.5% NaCl solution. J. Alloys Compd..

[59] Whartan J.A., Barik R.C., Kear G., Wood R.J.K., Stokes K.R., Walsh F.C. (2005). The corrosion of nickel–aluminium bronze in seawater. Corros. Sci..

[60] Kayvandarian F., Zanganeh D., Khodabakhshi F., Malekan M. (2024). Corrosion properties of submerged friction stir modified as-cast nickel-aluminum bronze (NAB) compositions. Electrochim. Acta.

